# *C9orf72* loss-of-function: a trivial, stand-alone or additive mechanism in C9 ALS/FTD?

**DOI:** 10.1007/s00401-020-02214-x

**Published:** 2020-09-02

**Authors:** Elke Braems, Bart Swinnen, Ludo Van Den Bosch

**Affiliations:** 1 Department of Neurosciences, Experimental Neurology, and Leuven Brain Institute (LBI), KU Leuven-University of Leuven, 3000 Leuven, Belgium; 2 Laboratory of Neurobiology, Experimental Neurology, Center for Brain and Disease Research, VIB, Campus Gasthuisberg, O&N4, Herestraat 49, PB 602, 3000 Leuven, Belgium; 3 Department of Neurology, University Hospitals Leuven, 3000 Leuven, Belgium

**Keywords:** C9orf72, Repeat expansion, Amyotrophic lateral sclerosis, Frontotemporal dementia, Loss-of-function, Neurodegeneration, Postmortem, In vitro, In vivo

## Abstract

A repeat expansion in *C9orf72* is responsible for the characteristic neurodegeneration in amyotrophic lateral sclerosis (ALS) and frontotemporal dementia (FTD) in a still unresolved manner. Proposed mechanisms involve gain-of-functions, comprising RNA and protein toxicity, and loss-of-function of the *C9orf72* gene. Their exact contribution is still inconclusive and reports regarding loss-of-function are rather inconsistent. Here, we review the function of the C9orf72 protein and its relevance in disease. We explore the potential link between reduced C9orf72 levels and disease phenotypes in postmortem, in vitro, and in vivo models. Moreover, the significance of loss-of-function in other non-coding repeat expansion diseases is used to clarify its contribution in *C9orf72* ALS/FTD. In conclusion, with evidence pointing to a multiple-hit model, loss-of-function on itself seems to be insufficient to cause neurodegeneration in *C9orf72* ALS/FTD.

## Introduction

A repeat expansion in the *C9orf72* gene is associated with two neurodegenerative disorders, which represent the extremes of a disease spectrum: amyotrophic lateral sclerosis (ALS) and frontotemporal dementia (FTD) [108]. In ALS, loss of motor neurons in the spinal cord, brainstem, and primary motor cortex causes muscle weakness. FTD is characterized by degeneration of cortical neurons in the frontal and temporal lobe resulting mainly in behavioral aberrations. Characteristic for both diseases is the large phenotypic variability, including the heterogeneous involvement of upper and/or lower motor neurons [90, 108]. In contrast, a common pathological hallmark in 97% of ALS and 45% of FTD patients is the occurrence of cytoplasmic inclusions containing the hyperphosphorylated and mislocalized RNA-binding protein TDP-43 [15, 69].

**Fig. 1:**
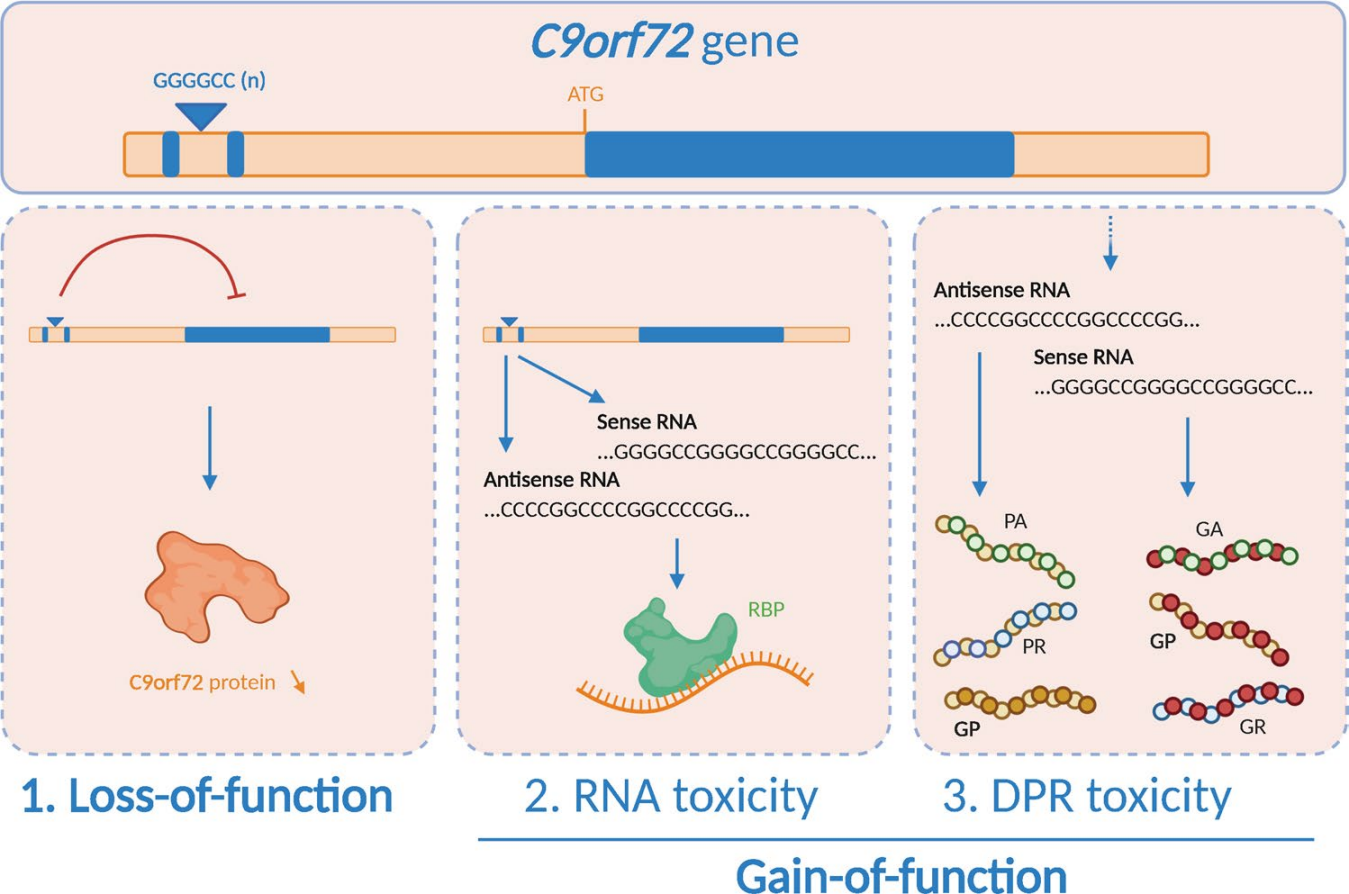
Three disease mechanisms proposed to underlie *C9orf72* ALS/FTD. First, the repeat expansion (GGGGCC)n could impede transcription processes leading to a reduction in C9orf72 protein levels and hence a loss-of-function of the gene. Second, through bidirectional transcription, the repeat expansion might form secondary sense (GGGGCC) and antisense (CCCCGG) RNA structures sequestering regulatory RNA binding proteins (RBPs). This process, called RNA toxicity, could induce a loss-of-function of these proteins. Third, the non-ATG repeat-associated (RAN) translation process forms potentially toxic dipeptide repeat proteins from the sense strand (GP, GA, GR) and the antisense strand (GP, PA, PR)

The ‘GGGGCC’ hexanucleotide repeat expansion in the *C9orf72* gene is the most common mutation in both familial (± 30%) and sporadic (± 5–10%) disease forms [28, 92, 114]. Despite extensive mechanistic research, the exact disease mechanism remains elusive. Three not mutually exclusive mechanisms have been proposed (Fig. 1) [43]. First, a gain-of-function (GOF) might result from repeat RNA sequestering functionally important RNA binding proteins. A second GOF mechanism is based on the observation of dipeptide repeat protein (DPR) species generated by repeat-associated non-ATG (RAN) translation of the GGGGCC repeat sequence. Third, a loss-of-function (LOF) mechanism might result from reduced C9orf72 protein levels.

Elucidating which (combination) of these mechanisms is the culprit for *C9orf72* ALS/FTD (C9 ALS/FTD) is crucial for two main reasons. On one hand, it might shed light on the disease mechanism of other monogenetic as well as sporadic forms of ALS/FTD. On the other hand, it is pivotal to tailor the appropriate treatment of C9 ALS/FTD. Several therapeutic approaches targeting one or more possible mechanisms have been proposed. However, their efficacy and safety still need to be demonstrated. So far, it is unclear whether targeting only one mechanism (e.g. DPR toxicity through DPR antibodies or LOF through *C9orf72* gene therapy) will be sufficient to have a disease-modifying effect in patients. Moreover, it is still debated whether targeting the GOF mechanisms using a knockdown approach (e.g. with antisense oligonucleotides (ASOs)) might have detrimental effects by exacerbating *C9orf72* LOF.

Whereas the contribution of the GOF mechanisms has already extensively been investigated and reviewed [37, 109] a critical appraisal of *C9orf72* LOF is currently lacking. In this review, we will provide an overview of the current knowledge on the physiological function of the C9orf72 protein, as well as the contribution of *C9orf72* LOF in *C9orf72* ALS/FTD pathogenesis.

## The physiological role of *C9orf72*

### The transcription and translation of *C9orf72*

**Fig. 2:**
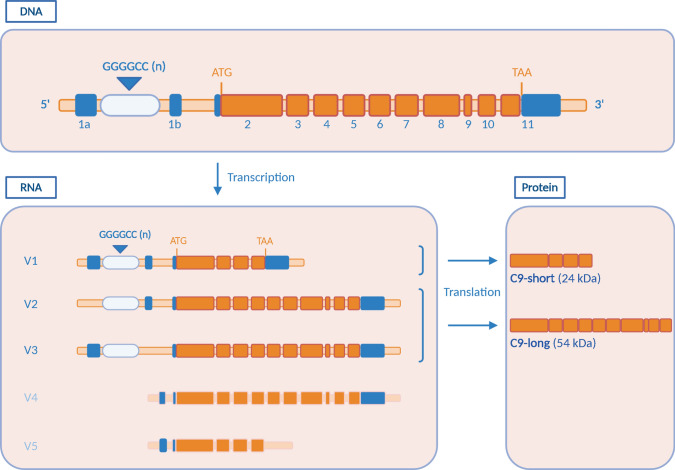
*C9orf72* gene structure, transcripts, and isoforms. The *C9orf72* gene contains 11 exons. Transcription of this gene results in three main transcripts (V1, V2, V3). The repeat region is located in the first intron of transcripts V1 and V3, whereas the V2 transcript contains the repeat in its promoter region. V4 and V5 are non-coding transcript variants with V5 being a truncated form of V4. Upon translation, two C9orf72 isoforms are formed. The short isoform (24 kDa) arises from the V1 transcript and the long isoform (54 kDa) is translated from V2 or V3

The *C9orf72* gene (Fig. 2) is located at the short arm of chromosome 9. Its sequence has a high degree of homology compared to different species commonly used as model systems (98.1% with *Mus musculus*, 97.7% with *Rattus norvegicus* and 76.0% with *Danio rerio* [26]). This suggests that the protein is involved in essential cellular processes.

At the mRNA level, diverse transcription start sites trigger the formation of three transcript variants (Fig. 2). Variant 1 (V1; NM_145005.7) and variant 3 (V3; NM_001256054.3), starting from exon 1a, contain the repeat expansion in the first intron, while variant 2 (V2; NM_018325.5) initiates its coding region from exon 1b and harbors the expansion in the promoter region [10, 30, 42]. In addition to these three conventional transcripts, at least two non-coding transcript variants (lncRNAs) have been described, including their natural antisense transcripts [30, 41, 93]. Due to the alternative transcription start sites, exon 1c is the first of transcript variant 4 (V4) and 5 (V5) with V5 representing a truncated form of V4 (Fig. 2). The contribution of these lncRNAs to the physiological function of C9orf72 and the C9 ALS/FTD disease mechanism has not yet been identified.

At the protein level, translation results in two isoforms: transcript variant 1 encodes a 222 amino acid isoform (called ‘C9-short’ or ‘C9-S’; 24 kDa), while transcript variants 2 and 3 encode a 481 amino acid isoform (called ‘C9-long’ or ‘C9-L’; 54 kDa) (Fig. 2).

### The cellular and subcellular distribution of *C9orf72*

At the cellular level, the highest *C9orf72* transcript expression is detected in a subset of myeloid cells [93], whereas protein levels (i.e. the long isoform) seem to be most abundant in the brain and spinal cord [38]. Because of the differential use of transcription start sites for *C9orf72* transcripts between myeloid and CNS tissues, C9orf72 function might differ between cell types [93]. In the central nervous system (CNS), *C9orf72* promoter activity is most abundant in neuronal and glial cell types recognized to degenerate in ALS/FTD which might render them more susceptible to the effects of *C9orf72* LOF as well as GOF toxicity [65]. Notably, quantitative mass-spectrometry-based proteomics revealed that C9orf72 has a 200-fold lower baseline expression compared to proteins like TDP-43 and FUS [31, 51], which also complicates the detection of endogenous C9orf72.

At the subcellular level, the highly soluble C9orf72 is detected in both the nucleus and the cytoplasm [38]. The long isoform is the most abundant one and mainly exhibits a diffuse nuclear distribution [123]. Both isoforms also exhibit a cytoplasmic vesicular distribution [33, 74], representing endosomes, lysosomes, and other autophagy-related vesicles, such as autophagosomes [33, 104]. In neurons, C9orf72 has also been localized to the presynaptic region [38].

### The protein function of *C9orf72*

#### Involvement of C9orf72 in membrane trafficking

To fully understand the underlying disease mechanism of *C9orf72* ALS/FTD, it is essential to elucidate the physiological function of C9orf72. Computational analyses revealed a structural homology between C9orf72 and DENN (differentially expressed in normal and neoplastic cells) proteins, implying C9orf72 to act as a RabGEF (Guanine Exchange Factor of Rab proteins) [68, 75]. These factors are well known to activate Rab GTPases (‘Rabs’), hence controlling membrane trafficking events (e.g. endocytosis and autophagy) in the cell. As C9orf72 also colocalizes with several of these Rabs, it is likely to have a function in membrane trafficking [33]. Association of C9orf72 with these Rab proteins indicates that C9orf72 may be specifically involved in endocytic and phagocytic trafficking [6, 33].

**Fig. 3:**
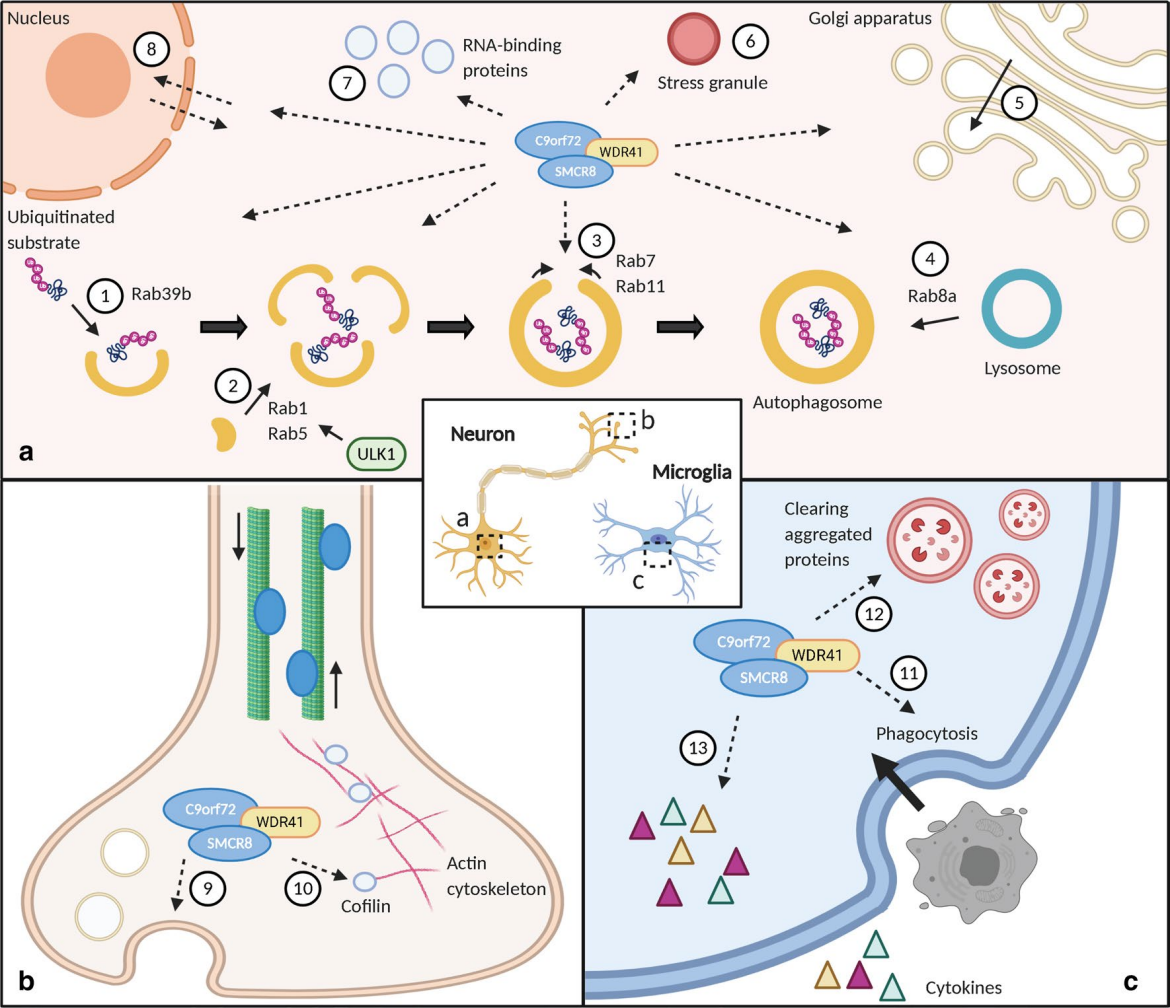
The function of C9orf72 in the central nervous system. **a** In neurons, C9orf72 (or tripartite complex C9orf72/SMCR8/WDR41) directly or indirectly regulates autophagy at four levels; recruitment of substrates to the phagophore (1), phagophore formation (2), maturation and closure of the autophagosome (3) and fusion of the autophagosome with lysosomes (4). Vesicle trafficking in the Golgi apparatus could also be controlled by C9orf72 (5). Interaction with stress granules (6), RNA binding proteins (7) and nucleocytoplasmic transport factors (8) points towards a possible regulating role of C9orf72 in phase separation (6), RNA metabolism (7) and nucleocytoplasmic transport (8). **b** C9orf72 also localizes presynaptically where it might interact with extracellular vesicle secretion (9) and the cytoskeleton (i.e. cofilin) (10). **c** In microglia, phagocytosis of dying neurons and other cells has been associated with C9orf72/SMCR8/WDR41 (11). C9orf72 is involved in the clearing of aggregated proteins (12) and the release of cytokines (13)

Disposing of cellular garbage (e.g. damaged organelles and other dysfunctional cellular components) is essential for cellular homeostasis and for recycling various factors and proteins. This process, called autophagy, can be mediated by several routes. One of these routes, macroautophagy, plays a role in neurodegeneration [39, 120]. One example is that depletion of essential autophagic factors in mice results in protein aggregation, motor neuron loss, and motor dysfunction [48, 60, 84]. C9orf72 forms a stable tripartite complex with WD repeat-containing protein 41 (WDR41) and Smith-Magenis chromosome region 8 (SMCR8) proteins [107, 117, 124]. This complex recruits Rab proteins and subsequently controls early stages of autophagy at four possible levels (Fig. 3a) [33, 101, 107]. A first level is the p62-mediated recruitment of ubiquitinated substrates to the phagophore through the interaction of Rab39b with the complex [84, 101, 110, 124]. Second, C9orf72 regulates the Rab1 and Rab5-dependent trafficking of the ULK1 autophagy initiation complex to induce autophagosome formation [4, 107, 117]. Third, the maturation and closure of the autophagosome by Rab7 and Rab11 might be regulated by C9orf72 [33]. Fourth, the interaction with Rab8a could trigger autophagosome-lysosome fusion [4]. Interestingly, C9orf72 and p62 colocalize with stress granules and Rab1a was identified to be part of these structures (Fig. 3a) [21, 54, 118]. Moreover, mass spectrometry revealed C9orf72 to interact with the heat-shock chaperone, HSC70, essential for the initiation of aggrephagy, a mechanism responsible for clearing aggregating proteins. Hence, it appears that C9orf72 might also be relevant in selective protein aggregation macroautophagy [84, 101]. Despite these rather descriptive associations with key autophagy players, the net impact of C9orf72 on autophagy is still unclear.

Next to its role in the initiation phase, C9orf72 seems to be involved in later phases of autophagy as well. Illustrating this, various *C9orf72* knockout models exhibit a general dysregulation of lysosomal homeostasis, including defects in lysosome morphology, lysosomal accumulation, and defective fusion of lysosomes to phagosomes [4, 5, 25]. Mouse studies indicate that C9orf72 regulates lysosomal exocytosis in macrophages and microglia [87, 127].

Based on work in *C. elegans* and neuroblastoma cell lines, C9orf72 was also associated with endocytic pathways [25]. C9orf72 interacts with Rab5 (part of early endosomes), Rab7 (part of late endosomes) and Rab11 (regulates recycling of endocytosed proteins), each associated with a critical phase in the formation of endosomes [50, 73, 129]. Next to autophagy initiation, lysosomes, and endosomes, C9orf72 could also be involved in other membrane trafficking events like extracellular vesicle secretion (Fig. 3b) and trans-Golgi vesicle trafficking (Fig. 3a) [6].

#### C9orf72 and the immune system

C9orf72 might also play a role in the immune system (Fig. 3c). This is mainly based on the observation of *C9orf72* knockout mice (as well as *SMCR8* knockout mice) developing inflammatory and autoimmune phenotypes, such as enlarged lymph nodes, splenomegaly, immune-mediated glomerulonephropathy and excessive release of inflammatory cytokines and autoantibodies [7, 16, 64, 79, 106]. These findings suggest that C9orf72 is involved in the immune homeostasis in macrophages and microglia [7, 87]. The exact underlying mechanism of this immune dysfunction is still unclear but might be related to the high expression level of *C9orf72* in myeloid cells. In addition, a link with the role of C9orf72 in membrane trafficking has been suggested. In the absence of C9orf72, microglia display a diminished ability to clear aggregated proteins [87, 93]. Moreover, defects in autophagy and lysosomal exocytosis have been observed in C9orf72 and SMCR8 double knockout mice [7, 64, 87, 107, 127].

#### Other functions of C9orf72

As mentioned before, C9orf72 has been reported to be involved in stress granules (Fig. 3a). In the event of stress signals, C9orf72 is recruited to stress granules [74]. The C9orf72 long isoform has been shown to be essential for the assembly of these granules [74]. More specifically, in the absence of C9orf72, cells become hypersensitive to stress signals, such as endoplasmic reticulum (ER) stress [74]. This might render the cells more vulnerable to toxicity induced by the products of gain-of-function mechanisms, including RNA foci and DPRs [66]. C9orf72 also localizes at the nuclear pore and interacts with components of the nuclear pore complex (Fig. 3a) [122]. Hence, C9orf72 might have a role in nucleocytoplasmic transport. Notwithstanding that nucleocytoplasmic transport defects are especially well studied in the context of gain-of-function mechanisms [11, 36, 59, 66, 115, 126], it remains ambiguous whether these defects could be related to or enhanced by *C9orf72* loss-of-function. Moreover, the interaction of C9orf72 with cofilin and other actin binding proteins [105] indicates that C9orf72 could be involved in the organization of actin filaments in neuronal axons (Fig. 3b). Last but not least, RNA binding proteins (e.g. hnRNPK, hnRNPA3…) are associated with C9orf72, suggesting that, next to their role in RNA toxicity, these proteins might also be involved in the LOF mechanism (Fig. 3a) [24, 47, 83].

## Assessment of the loss-of-function mechanism in *C9orf72* ALS/FTD

To assess the contribution of *C9orf72* LOF in C9 ALS/FTD, we will address two main aspects. On one hand, the impact of the repeat expansion on C9orf72 physiology (i.e. epigenetics, transcription, protein levels et cetera) and functionality (i.e. downstream targets) in C9 ALS/FTD patients needs to be assessed. On the other hand, determining whether this degree of impact is sufficient to induce (or contribute to) neurodegeneration is crucial as well.

### Postmortem studies

#### Homozygous C9orf72 patients and coding mutations in C9 ALS/FTD

Classical genetic indications of a LOF mutation include increased clinical severity in the case of homozygosity of the mutation, as well as mutations occurring in the coding region (e.g. nonsense mutations or missense mutations). Although very rare, two independent studies each reported one patient homozygous for the *C9orf72* repeat expansion. These patients did not exhibit a more severe clinical phenotype compared to heterozygous cases [23, 35]. Moreover, no coding mutations in the *C9orf72* gene were found in C9 ALS/FTD [49], except for one solitary patient harboring a splice site mutation in exon 5 [71]. Nonetheless, a neurological clinical examination, autopsy, and assessment of a *C9orf72* repeat expansion have not been performed in this patient.

#### Epigenetics

Epigenetic modifications are known to alter gene expression through e.g. DNA methylation and histone modifications [46]. Several studies investigated methylation levels of the *C9orf72* promoter and the repeat expansion itself in genomic DNA from the patient brain and/or blood samples [8, 45, 53, 70, 80, 94, 121]. Hypermethylation of CpG sites in the promoter region of the *C9orf72* gene, leading to decreased promoter activity, is associated with shorter disease duration (i.e. a more aggressive disease course) [45, 53, 70], supporting LOF. However, other studies contradict this finding and suggest an association of *C9orf72* promoter hypermethylation with increased survival [80, 94]. As the majority of these studies only investigated methylation patterns of the promoter region, uncertainty about a protective or detrimental effect might be explained by additional hypermethylated regions elsewhere in the gene. Next to the promoter, the *C9orf72* repeat expansion itself can be hypermethylated [121], which is the case in almost all (97%) repeat expansion carriers, whereas only one third has a hypermethylated *C9orf72* promoter region [70]. As hypermethylated patterns can spread along a gene [58], this suggests that the epigenetic alteration originated in the repeat region and subsequently spread towards the promoter region. Moreover, methylation can be seen as a neuroprotective response by preventing expression of the *C9orf72* repeat expansion and hence suppressing the formation of potentially toxic RNA foci and/or DPRs [80, 121].

#### Transcript levels

Several studies evaluated *C9orf72* transcript levels in postmortem tissue, examining the total *C9orf72* transcript level and/or individual transcript levels of V1 and V3, containing the repeat in the first intron, and V2, harboring the repeat in the promoter region (Table 1) [9, 10, 17, 22, 28, 34, 44, 53, 82, 116]. The frontal cortex, cerebellum, and lumbar spinal cord are the most frequently analyzed CNS regions in these studies. Considering all reports, an overall decrease (~ 50%) in total sense *C9orf72* transcript levels in frontal cortex and cerebellum is evident and, interestingly, independent of age-at-onset or age-at-death [9, 10, 22, 34, 44, 82, 116]. However, measuring individual transcripts reveals that V3 remains unaltered, while V1 and especially V2 levels are significantly decreased [10, 28, 116]. Moreover, similar reductions in V1 and V2 were observed in blood samples [53]. The obvious decrease of V2 levels in both blood and CNS samples is probably due to the repeat being located in its promoter region. The repeat expansion might inhibit the binding and/or function of polymerases and transcription initiation factors. V1 and V3 comprise the expansion in the first intron and might, therefore, be less susceptible to transcriptional defects.

**Table 1:**
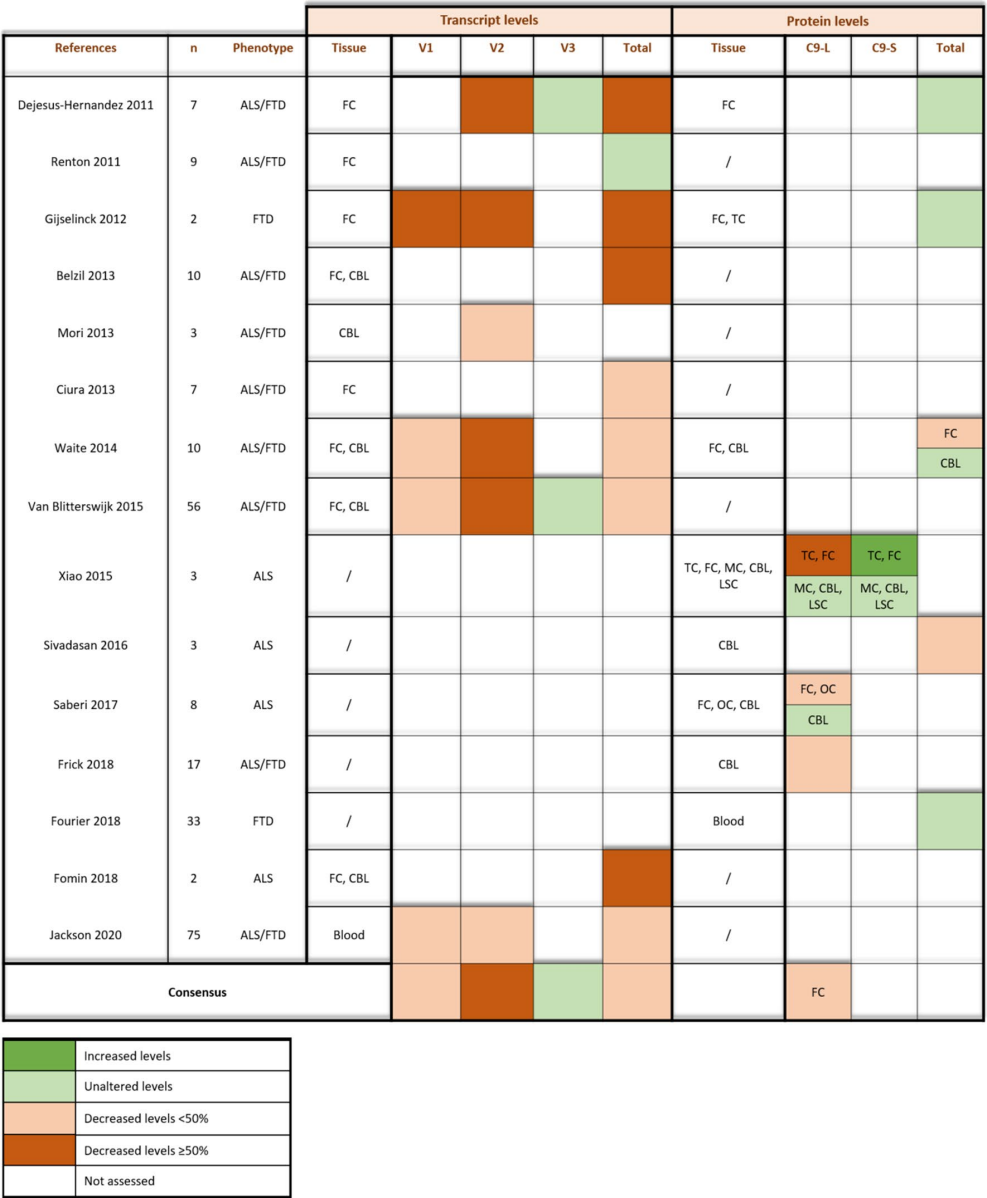
Overview of postmortem *C9orf72* transcript and protein levels

Assessment of *C9orf72* transcript and protein levels in *C9orf72* ALS/FTD patient material. For each study, the amount of included cases (*n*) and analyzed tissues is denoted. The overall consensus of the different studies is indicated. Dark green, increased; light green, unaltered; orange, < 50% decrease; brown, ≥ 50% decrease

*CBL* cerebellum, *FC* frontal cortex, *TC* temporal cortex, *MC* motor cortex, *OC* occipital cortex, *LSC* lumbal spinal cord

#### Protein levels

Decreased transcript levels do not necessarily implicate decreased protein levels (Table 1). In the frontal cortex of C9 ALS/FTD patients, a 25% decrease (compared to healthy controls, sALS cases and neurodegenerative disease controls) of the long protein isoform C9orf72 was consistently observed [95, 116, 122]. Measurements of cerebellar protein levels are rather inconsistent, ranging from 80% reduction [38, 105] to unaltered protein levels [95, 116, 122]. Moreover, the reduced cerebellar levels do not associate with clinical findings, questioning the disease relevance of cerebellar protein levels [38]. Altogether, a rather modest (25%) decrease of C9orf72 protein levels seems to be rather small to support a LOF mechanism. However, one needs to keep in mind that protein levels might be lower in disease-relevant cell types (e.g. affected neurons) but might escape detection due to a dilution effect. Also, disease-relevance of the examined tissue in most studies is not so straightforward as the denominator ‘frontal cortex’ encompasses both the prefrontal cortex as well as the primary motor cortex, the former being more relevant in FTD and the latter in ALS. Additionally, issues regarding C9orf72 antibody specificity and small sample size further complicate this matter. C9orf72 protein levels do not seem to correlate with age [38]. On the other hand, patients with large repeat expansions show an increased expression of the C9orf72 short isoform, whereas intermediate repeat carriers contain a higher expression of the long isoform [17, 122]. This suggests a possible repeat length-dependent effect on C9orf72 expression level and isoform distribution. Hence, the presence of repeat mosaicism (i.e. different *C9orf72* repeat sizes in different tissues) between and within patients might obscure the effect on the expression level of the disease-relevant protein products.

#### Conclusions

Altogether, genetic and epigenetic data are difficult to reconcile with a predominant LOF mechanism. Most importantly, while C9orf72 transcript levels seem to be reduced by approximately 50%, protein levels are probably reduced by only 25% and there is no indication that C9orf72 functionality is impaired in C9 ALS/FTD patients. As a consequence, further research is required. Specifically, assessment of all individual transcript levels, the contribution of long *versus* short protein isoform, development of specific antibodies, increased sample sizes, and assessment of disease-relevant brain regions and cell types will be indispensable.

### In vitro studies

Whereas postmortem material can be regarded as the end-stage of the disease mechanism, in vitro models with a disease-relevant phenotype might be able to provide earlier mechanistic insights. Additionally, various knockdown approaches (i.e. siRNA, antisense oligonucleotides, CRISPR/Cas9) can be harnessed to assess whether *C9orf72* LOF could be harmful.

#### C9 patient-derived models

##### Epigenetics

An asset for the use of iPSC-derived neurons as a model system is that the *C9orf72* promoter hypermethylation observed in patients is reacquired upon neuronal differentiation [32]. Therefore, this model might be used to study the role of epigenetics in C9 ALS/FTD. In patient lymphoblastoid cell lines, the demethylation of *C9orf72* promoter (associated with increased gene expression) increases the cell vulnerability to oxidative and autophagic stress [70]. This implies that the observed hypermethylation probably represents an epigenetic silencing as a protective regulatory reaction to transcriptional instability due to the hexanucleotide repeat expansion. As a consequence, epigenetic in vitro data are difficult to reconcile with a predominant LOF mechanism.

##### Transcript and protein levels

In keeping with postmortem findings (cf. supra), most C9 patient iPSC-derived neurons exhibit an overall reduction (50%) in *C9orf72* transcripts (Table 2) [3, 29, 78, 104, 117]. On the contrary, a recent study did not observe these changes in individual and total transcript levels [40]. Also, fibroblasts and iPSCs do not show these consistent transcript alterations (Table 2) [3, 29, 40, 98]. Moreover, protein levels seem to be reduced in fibroblasts, but not in C9 iPSC-derived motor neurons (MNs) (Table 2) [1, 92, 98, 102]. As a consequence, there are discrepancies between transcript and protein alterations between models and decreased *C9orf72* transcript levels do not always correlate with decreased C9orf72 protein levels. Issues regarding C9orf72 antibody specificity could contribute to this discrepancy [28, 92]. Numerous C9orf72 antibodies (including polyclonal, monoclonal and recombinant) have been developed, but remain largely unverified. Interestingly, a novel powerful CRISPR genome-editing approach validated multiple commercially available C9orf72 antibodies, which could be of use and may be considered for future *C9orf72* loss-of-function studies [62].

**Table 2:**
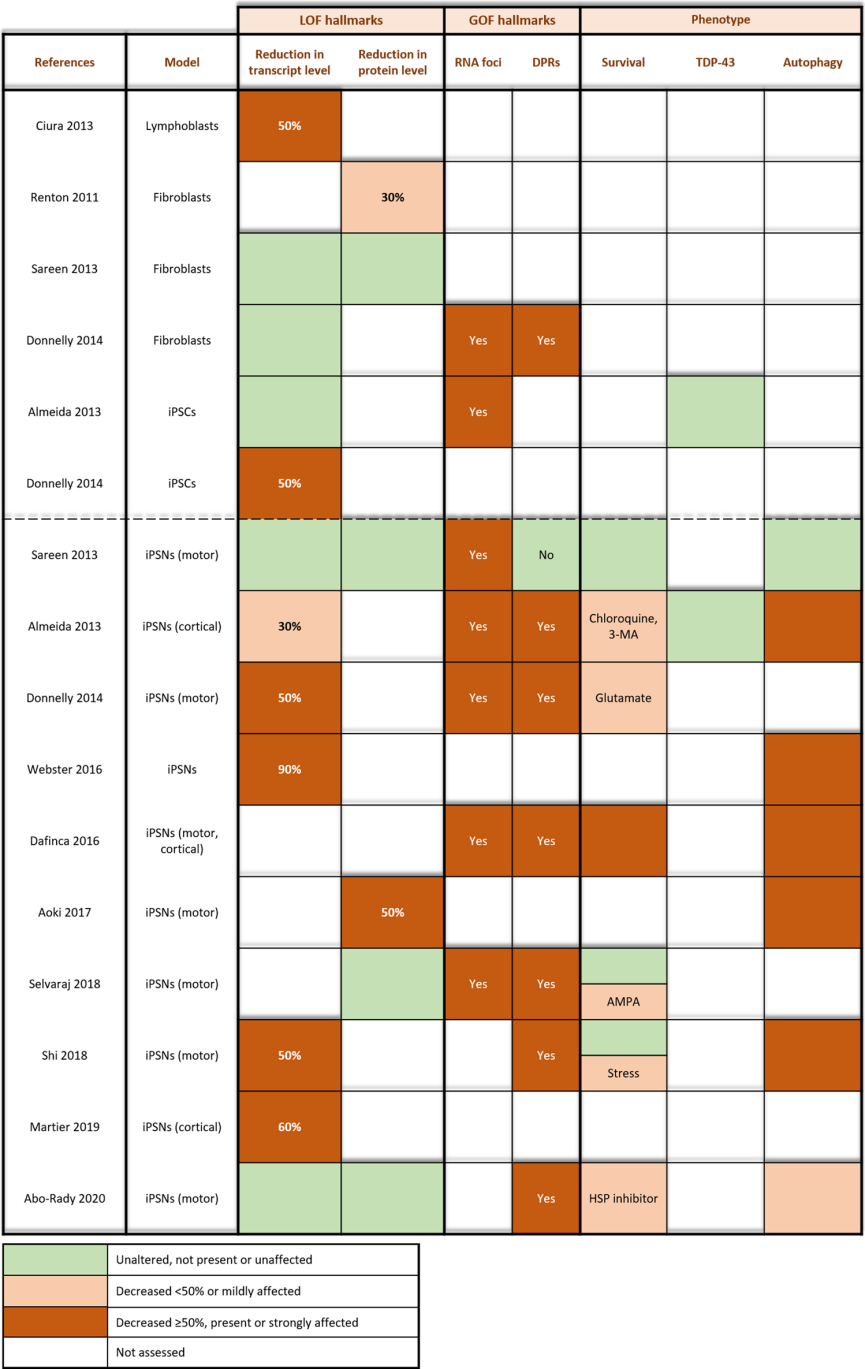
Overview of *C9orf72* ALS/FTD patient-derived in vitro models

Assessment of *C9orf72* transcript and protein levels in C9 ALS/FTD patient-derived models (i.e. fibroblasts, lymphoblasts, and iPSC-derived neurons (iPSNs)). The presence of hallmarks for RNA toxicity (i.e. RNA foci) and DPR toxicity (i.e. DPR proteins) is denoted for each study. Altered survival, TDP-43 pathology and autophagy impairment are indicated as well. Legend: green, unaltered, not present or unaffected; orange, mildly decreased (< 50%) or affected; brown, strongly decreased (≥ 50%), present or strongly affected

##### Impaired autophagy

In C9 patient iPSC-derived MNs, several hallmarks of impaired autophagy were observed, including lysosomal alterations, p62 aggregation, proteasomal degradation, and reduced autophagic flux (Table 2) [1, 3, 6, 27, 104, 117]. However, it is unclear so far whether this autophagic disturbance is caused by the GOF or LOF mechanism as these cells contain the ‘GGGGCC’ repeat expansion. Furthermore, a synergistic mechanism is not excluded as autophagy is needed to clear toxic GOF products (i.e. DPRs) [1, 12, 88].

#### Modelling *C9orf72* knockdown and knockout

Wild type models (i.e. in the absence of a GGGGCC repeat expansion) have the advantage of evaluating the effect of *C9orf72* knockdown without confounding GOF mechanisms. This allows a clearer evaluation of the effect of *C9orf72* knockdown. However, one needs to keep in mind that these models are artificial, hence, disease relevance might be lacking. Therefore, efforts should be done to make these models as reminiscent as possible to C9 patient-derived models. Methods including siRNA, shRNA, and ASOs to knock down or CRISPR/Cas9 to knock out *C9orf72* were used to model and investigate the *C9orf72* LOF hypothesis in vitro (Table 3). Whereas most knockdown approaches do not or only partially (30%) affect C9orf72 protein levels, knockout models represent a full ablation of the protein. Importantly, reduction in transcript levels do not correlate with a similar reduction in protein levels. To illustrate this, a siRNA-mediated reduction of 50% in total transcript levels does not affect protein levels in primary neuronal cultures [101], whereas an ASO-induced 90% transcript reduction only corresponds to a 30% decrease in protein level in iPSC-derived neurons (iPSNs) [98]. A number of reasons could explain the discrepancy between transcript and protein levels [72], including technical biases that account for different quantitation methods. Additionally, post-translational modifications, half-life of C9orf72 proteins, and the fact that levels often represent a snapshot in disease progression are reasons to consider the lack of transcript-protein level correlation even more critically.

**Table 3:**
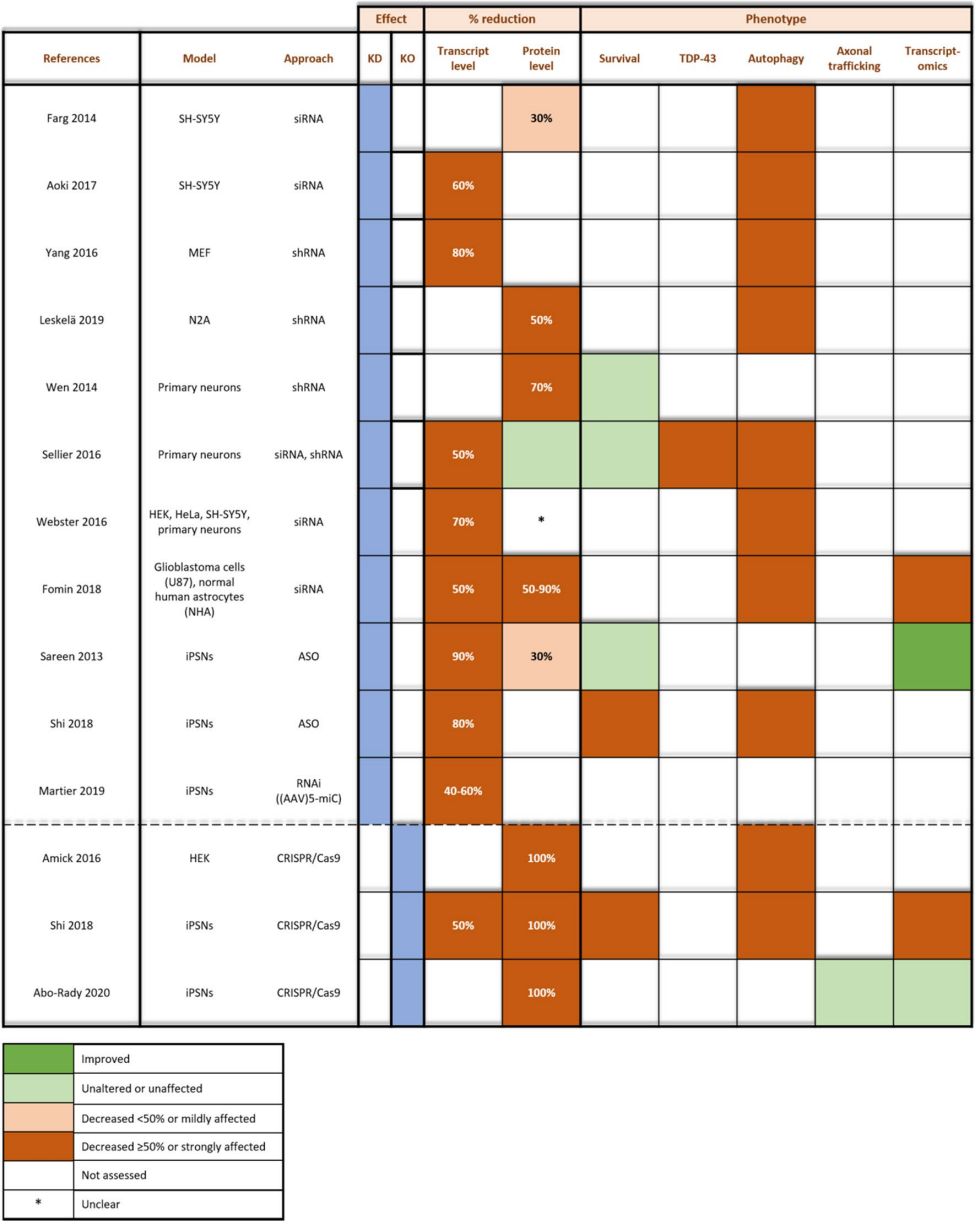
Overview of in vitro* C9orf72* knockdown (KD) and knockout (KO) models

Assessment of transcript and protein levels and corresponding phenotype in *C9orf72* knockdown/knockout cell models. Observed phenotypes (i.e. survival analysis, TDP-43 aggregation, autophagy deficits, axonal trafficking, and altered gene expression patterns) are indicated per study. Legend: dark green, improved; green, unaltered or unaffected; orange, mildly decreased (< 50%) or impaired; brown, strongly decreased (≥ 50%) or affected; white, not assessed

*Unclear results

##### Effect of C9orf72 depletion on neuronal survival

In most studies, survival of iPSNs and primary cortical neurons is largely unaffected upon reduction of *C9orf72* (Table 3) [98, 101, 119]. However, heterozygous and homozygous *C9orf72* deletion (as well as ASO-mediated knockdown) induces decreased survival of iPSNs in one study [104]. Exogenously adding C9orf72 rescues the survival of these neurons, indicating that C9orf72 depletion might play a role in the observed neurodegeneration in patients [104]. Interestingly, mutations in the ALS-related Ataxin-2 gene synergizes with the effect of *C9orf72* knockdown on motor neuron dysfunction and cell death [101]. This is in favor of a multiple-hit mechanism, since lowering *C9orf72* transcript levels on itself is not deleterious. TDP-43 pathology (i.e. a hallmark of *C9orf72* ALS/FTD) was assessed in only one study, in which increased TDP-43 accumulation was reported upon knockdown of *C9orf72* [101].

##### Effect of C9orf72 depletion on gene expression

An overall depletion of *C9orf72* (i.e. 90–100% protein reduction) results in transcriptome alterations in different cell types (Table 3) [34, 104]. Amongst others, alterations in immune system pathways, endothelin signaling, and multiple glutamate cycling genes were observed [34]. Interestingly, gene expression changes observed in both heterozygous and homozygous knockout models are similar to those observed in postmortem tissue from C9 ALS/FTD patients [104]. In contrast to these findings, gene expression profiles improve upon partial knockdown [98] or remain unaltered upon knockout of *C9orf72* [1]*.* Improved sequencing techniques (e.g. single-cell analyses) might be needed to clarify these discrepancies.

##### Effect of C9orf72 depletion on autophagy and other C9orf72-related processes

As expected from the supposed physiological function of C9orf72 (cf. supra), autophagic [6, 34, 67, 101, 117, 124], lysosomal [5, 6, 104] and endosomal [33] dysfunction was reported in in vitro* C9orf72* knockdown and knockout models (Table 3). However, lysosomal transport in axons of MNs lacking C9orf72 is similar to control lines, whereas this trafficking is clearly impeded in C9 patient iPSC-derived MNs [1, 88]. Interestingly, C9 depletion in the latter exacerbates the phenotype, suggesting a synergistic mechanism with GOF.

#### Conclusions

In patient-derived models, the most important observation is the presence of autophagic alterations. Given the physiological role of C9orf72, these disturbances might be caused by *C9orf72* LOF. However, this autophagic dysfunction has not been demonstrated to be disease-relevant and might as well be caused by the GOF mechanisms. Findings in *C9orf72* knockdown models further corroborate the role of C9orf72 in autophagy. However, apart from one report, *C9orf72* knockdown does not seem to induce in vitro neurotoxicity. Other ALS hallmarks (like TDP-43 pathology) were mostly not assessed. Interestingly, both types of in vitro models provide a hint towards a synergistic mechanism between LOF and GOF, which needs to be elaborated further.

### In vivo studies

As the interplay between different cell types clearly contributes to the ALS/FTD disease mechanism and progression, in vivo studies are of uttermost importance to elucidate pathways. Lower order organisms, such as *C. elegans* and *Danio rerio*, expanded our knowledge on the *C9orf72* ALS/FTD mechanism (Table 4). However, data validation and more sophisticated studies are needed in higher-order organisms, such as mice and rats.

**Table 4:**
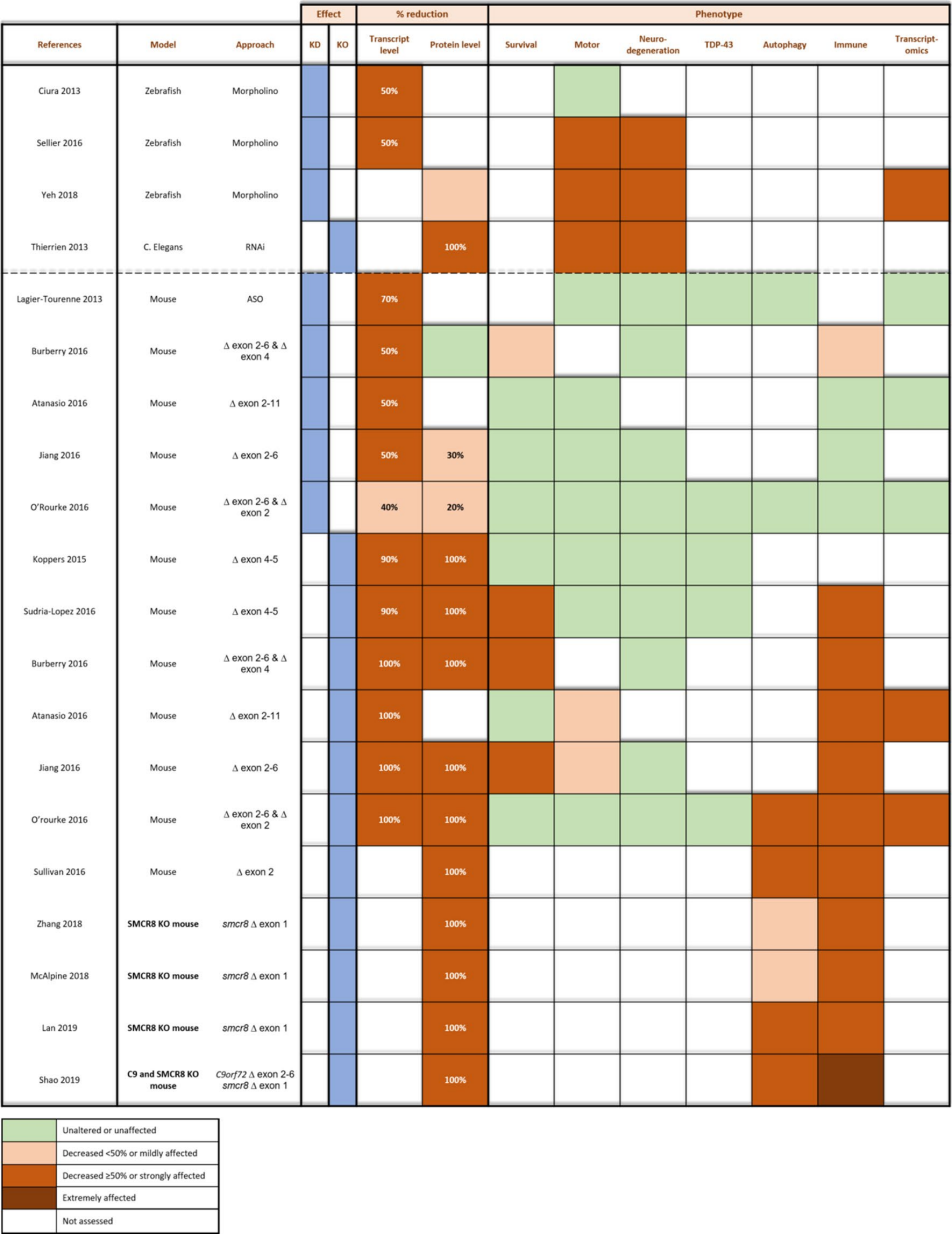
Overview of in vivo* C9orf72* knockdown (KD) and knockout (KO) models

Assessment of transcript and protein levels and corresponding phenotype in *C9orf72* knockdown/knockout *C. elegans*, zebrafish, and mouse models. Observed phenotypes (i.e. survival analysis, motor and neurodegenerative phenotype, TDP-43 aggregation, autophagy deficits, immune phenotype, and altered gene expression patterns) are indicated per study. Legend: green, unaltered or unaffected; orange, mildly decreased (< 50%) or impaired; brown, strongly decreased (≥ 50%) or affected; dark brown, extremely affected; white, not assessed

#### Modelling C9orf72 loss-of-function in lower-order organisms

The first in vivo* C9orf72* null mutation was generated in the invertebrate *C. elegans* [113]. Age-dependent motor neuron degeneration and stress sensitivity was observed upon deleting the C9 orthologue [113]. Moreover, these defects synergized with toxicity caused by mutant TDP-43 proteins, being in favor of a combined LOF and GOF mechanism [113].

To evaluate the effect of C9orf72 on the developing nervous system in a developmental model and addressing the LOF hypothesis in a vertebrate model, a morpholino-mediated knockdown (50% reduction in transcript level) of *C9orf72* was performed in zebrafish embryos [22, 125]. This downregulation induces both cellular (i.e. axonal length and embryo morphology) and behavioral deficits (i.e. swimming distance and velocity) associated with locomotion [22, 125]. Although these findings indicate that C9orf72 is crucial for neuronal development, transgenic zebrafish lines with a stable *C9orf72* knockdown are essential to validate these results.

#### Modelling loss-of-function in higher-order organisms

More mechanistic studies were performed in *C9orf72* knockdown and knockout mouse models (Table 4). First, **knockdown** of *C9orf72* induces a 50–70% reduction of *C9orf72* RNA, without a decrease in protein level [7, 16, 63]. No behavioral or motor phenotypes are observed. Furthermore, C9orf72 hallmarks including TDP-43 pathology and p62 or ubiquitin aggregates are absent. This is in line with findings in cellular models indicating that reduced RNA levels are insufficient to cause motor neuron disease. Second, **knockout** mouse models allow long-term observation and analysis of acute and chronic effects of C9orf72 depletion. Remarkably, these models are mainly characterized by the lack of motor neuron degeneration and the presence of immune phenotypes [16, 55, 61, 87, 104, 106, 107].

##### Immune dysfunction, but no motor phenotype in C9orf72 KO mice

Neuronal-specific depletion of C9orf72 induces a decrease in body weight, without motor impairment or decreased survival [61]. Similarly, mice lacking C9orf72 in all tissues exhibit decreased body weight and immune dysfunction without motor neuron impairments or degeneration [7, 16, 55, 87, 96, 106, 128]. Moreover, in contrast to the *C9orf72* KD in primary cortical neurons (cf. supra) [101], no TDP-43 pathology is present in these mouse models [61, 106]. Notably, a possible mild motor phenotype (i.e. weakness of hindlimbs, but no differences on rotarod) has been detected in one study [7]. However, this finding might constitute a secondary effect as the immune phenotype already appeared before the motor symptoms. The immune phenotype comprises splenomegaly, enlarged lymph nodes, infiltration of immune cells, increased expression of inflammatory cytokines, presence of autoantibodies and immune-mediated glomerulonephropathy [7, 16, 55, 106]. In line with these observations, RNA sequencing reveals gene dysregulation in inflammatory pathways [7, 87].

##### Lysosomal and autophagy-related alterations in C9orf72 KO mice

C9orf72-deficient mice exhibit autophagy defects in the spleen and liver, which are accompanied by increased lysosomal proteins and increased autophagy initiation (Table 4) [87, 107]. Mice deficient in SMCR8, a factor in the tripartite complex with C9orf72, display similar autophagy defects as well as inflammatory and autoimmune phenotypes (Table 4) [64, 79, 103, 127]. Moreover, a combined knockout of *C9orf72* and *SMCR8* induces even more severe immune disturbances (Table 4) [103]. This confirms that both factors act in the same pathway and are partially redundant upon individual depletion. Strikingly, knocking out one or both *C9orf72* alleles in a mouse model characterized by the C9orf72 ALS hallmarks (i.e. RNA foci, DPRs), prevents the likely protective activation of autophagy, which is driven by the presence of DPRs [128]. Moreover, it worsens the neuronal loss and cognitive phenotype [128]. Once more, this implies that a combination of loss- and gain-of-function is at play.

#### Conclusions

The motor phenotype in zebrafish and *C. elegans* is in contrast to the findings in C9 LOF mouse models, which display an immunological phenotype but no motor neuron degeneration. The phenotypic discrepancy might be explained by a difference in the homology of the human *C9orf72* orthologues in the various disease models (i.e. 21% in *C. elegans*, 75% in zebrafish and 98% in mice) and differences in RNA and protein processing events (i.e. splicing, post-translational modifications). Moreover, findings in the C9 LOF mice could imply that in case of a primary LOF mechanism, patients should present with some immune dysfunction and/or autoimmunity. Apart from one study showing a 12% prevalence of nonthyroid autoimmune disease in a small cohort of C9 ALS/FTD patients [81], there are currently no studies reporting immunological issues in C9 ALS/FTD. Most importantly, whereas the immune phenotypes occur in mice deficient of C9orf72 (i.e. knockout), this is not the case when C9orf72 is only partially reduced (i.e. knockdown). As C9 ALS/FTD patients also exhibit a partial (at best 25% in protein level) reduction, this does not seem to be in line with a prevailing LOF mechanism.

## Loss-of-function in other non-coding repeat expansion disorders

In non-coding repeat expansion disorders, a specific short DNA sequence is repeated multiple times. These disorders exhibit considerable phenotypic and pathogenic differences, probably depending on the genomic location (i.e. promoter region, intron, 5′ and 3′ untranslated regions (UTR)) as well as the sequence of the repeat expansion. Understanding the contribution of LOF mechanisms in the neurodegenerative and neurodevelopmental disorders caused by non-coding repeat expansions might elucidate the likelihood of *C9orf72* LOF playing a role in C9 ALS/FTD (Table 5). Here, we will discuss the non-coding repeat expansion disorders in which LOF can be considered.

**Table 5:**
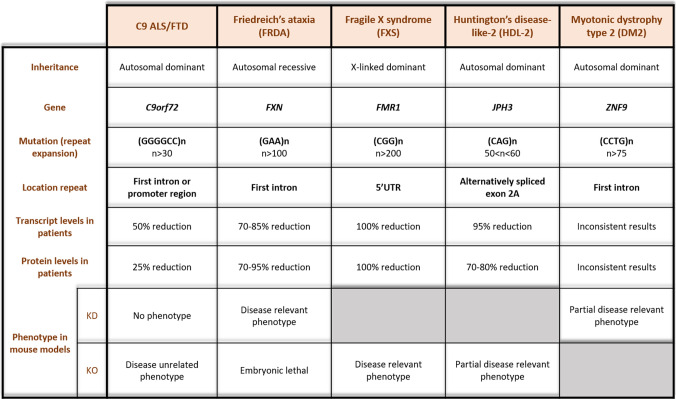
Overview of loss-of-function contribution in non-coding repeat expansion disorders

Genetic features comprising gene name, function, repeat expansion, and location are indicated. Patient transcript and protein levels of the disease-related gene are summarized in combination with the observed phenotype in the corresponding knockdown (KD) or knockout (KO) mouse models. References: C9 ALS/FTD, see Table 1, 2 and 3 [28, 92, 108]; FRDA, [18, 19, 77, 99]; FXS, [52, 56, 86, 112]; HDL-2, [76, 85, 100]; DM2, [14, 20, 91]

### Friedreich’s ataxia (FRDA)

The autosomal recessive neurodegenerative disease FRDA, characterized mainly by ataxia and degeneration of the spinocerebellar tract, is caused by a GAA expansion in the first intron of the *FXN* gene [2, 18, 77, 99]. In patients, an overall reduction of 70–85% in *FXN* transcript levels and 70–95% in frataxin protein levels are observed in the cerebellum [18]. *FXN* KO mouse models are embryonically lethal, whereas the inducible hemizygous *FXN* mice recapitulate hallmarks of FRDA (severe ataxia, mitochondrial and cardiac abnormalities) [19]. Given the recessive inheritance, significantly reduced protein levels in patients and FRDA-like phenotype in *FXN* hemizygous KO mice, it is believed that LOF is the primary mechanism of FRDA [77, 99].

### Fragile X syndrome (FXS)

*FMRP translational regulator 1 (FMR1)* harbors a trinucleotide repeat (i.e. CGG) in its 5′UTR region [56]. When this repeat is expanded above 200 repeats, it induces the neurodevelopmental syndrome FXS characterized by learning disabilities and cognitive impairment. Shorter (60 < *n* < 200) repeat expansions cause the neurodegenerative Fragile X-associated tremor/ataxia syndrome (FXTAS), in which increased repeat-containing *FMR1* transcripts are believed to cause a toxic RNA GOF [13, 111]. In FXS the long repeat expansion (*n* > 200) induces silencing of the *FMR1* gene (through hypermethylation), leading to an obvious LOF [56, 86, 89, 112]. This is supported by the observation of an *FMR1* KO mouse model recapitulating all hallmarks of FXS patients [52].

### Huntington’s disease-like-2 (HDL-2)

HDL-2 resembles Huntington’s disease both clinically (movement disorder and cognitive decline) and pathologically (intranuclear inclusions) [76]. It is caused by a CAG repeat expansion (*n* = 50–60) in the alternatively spliced exon 2A of the *junctophilin 3* (*JPH3)* gene [76]. This gene encodes a component of junctional complexes between the ER and plasma membrane. The mechanism underlying the autosomal dominant HDL-2 is mainly RNA and RAN toxicity consisting of toxic inclusions [76]. However, as transcript (~ 90%) and protein (~ 70–80%) decreases have been observed in the frontal cortex of patients [100], the contribution of a LOF mechanism is not excluded. Notably, a *JPH3* KO mouse model exhibits a motor phenotype. However, neurodegeneration or intranuclear inclusions have not been observed [85, 100].

### Myotonic dystrophy type 2 (DM2)

The autosomal dominant neuromuscular disorder DM2, characterized by progressive muscle wasting and weakness, is caused by a CCTG repeat expansion in the first intron of the *Zinc Finger Protein 9* (*ZNF9) *gene [14]. RNA toxicity is the predominant mechanism and is mediated by the sequestration of MBNL leading to aberrant splicing of several genes [57, 97]. There is no consensus on altered *ZNF9* transcript and protein levels [14, 91]. Apart from a partial KO of *ZNF9* in mice reflecting some of the aspects of DM2, including heart conductivity abnormalities and myotonic deficits [20], there is little evidence for a LOF mechanism to be involved in DM2.

Overall, non-coding repeat expansion disorders with LOF as principal disease mechanism (i.e. FRDA and FXS) display a 70–100% reduction of the respective transcript and protein levels and knockout mouse models recapitulate clinical and pathological phenotypes of patients (Table 5). This is in clear contrast with C9 ALS/FTD, where protein reduction (25%) is rather limited and KO mouse models lack a motor phenotype and neurodegenerative hallmarks. More related to C9 ALS/FTD, in HDL-2 and DM2, multiple lines of evidence imply GOF (RNA toxicity, RAN toxicity) as a primary mechanism with a lesser contribution of LOF. Altogether, comparing non-coding repeat expansion disorders based on their inheritance pattern, transcript, and protein level in patients, location of the repeat region, and knockout mouse models suggests a predominant GOF mechanism in C9 ALS/FTD.

## Conclusions

With the uprising evidence for GOF (i.e. RNA toxicity and DPR toxicity) mechanisms in C9 ALS/FTD, the contribution of a *C9orf72* LOF is still debated. Inconclusive findings regarding transcript and protein levels, functionality, and the correlation with an ALS/FTD phenotype in patients and different LOF model systems complicate this assessment. Nevertheless, in vitro and in vivo models made clear that the extent of *C9orf72* transcript and protein level reduction observed in patients seems to be insufficient to cause neurodegeneration in these models. Together with the findings inferred from other non-coding repeat expansion disorders, we conclude that *C9orf72* LOF is unlikely to be the main culprit in C9 ALS/FTD. Nevertheless, a synergistic mechanism in which *C9orf72* LOF might exacerbate the GOF mechanisms is not excluded and multiple recent studies even support such synergy. In the event of this multiple-hit mechanism, a therapeutic strategy to target both mechanisms will be even more challenging to develop. Nonetheless, targeting one of these mechanisms could be enough to prevent accumulative events causative for neurodegeneration. Either way, all these insights highlight the importance of studies taking into account, not one, but multiple C9 ALS/FTD mechanisms.

## Abbreviations

ALS: Amyotrophic lateral sclerosis
ASO: Antisense oligonucleotide
CNS: Central nervous system
DENN: Differentially expressed in normal and neoplastic cells
DPR: Dipeptide repeat protein
ER: Endoplasmic reticulum
FTD: Frontotemporal dementia
GEF: Guanine exchange factor
GOF: Gain-of-function
iPSC: Induced pluripotent stem cell
iPSN: IPSC-derived neuron
KD: Knockdown
KO: Knockout
LOF: Loss-of-function
MN: Motor neurons
RAN: Repeat-associated non-ATG
RBP: RNA binding protein
SMCR8: Smith–Magenis chromosome region 8
UTR: Untranslated region
WDR41: WD repeat-containing protein 41
